# Interplay of Vestibular Symptoms and Intracranial Hypertension: A Diagnostic Challenge in a Patient With Tullio's Phenomenon and Elevated Intracranial Pressure

**DOI:** 10.7759/cureus.89827

**Published:** 2025-08-11

**Authors:** Maria Belen Solis, Joseph Martinez, Relfa Proano, Hector A Lalama

**Affiliations:** 1 Neurology, Larkin Community Hospital, Miami, USA; 2 Medical School, Ross University School of Medicine, Miami, USA

**Keywords:** benign paroxysmal positional vertigo (bppv), intracranial hypertension, papilledema, tullio’s phenomenon, vestibular rehabilitation, vestibular symptoms

## Abstract

This case report describes a 45-year-old Hispanic female with a history of hypertension who presented with persistent, debilitating dizziness exacerbated by loud noises that began approximately two weeks ago. She was initially misdiagnosed with sinusitis and treated with antibiotics and decongestants for 10 days. A comprehensive neurological evaluation, including vestibular evoked myogenic potential (VEMP) testing and magnetic resonance imaging (MRI), revealed findings consistent with benign paroxysmal positional vertigo (BPPV) and a possible component of superior semicircular canal dehiscence (SSCD). MRI also raised concern for intracranial hypertension, with evidence of optic nerve tortuosity, a partially empty sella turcica, and papilledema confirmed with fundoscopy. Audiologic testing indicated left-sided mild sensorineural hearing loss, and the Dix-Hallpike maneuver confirmed positional vertigo. The patient experienced marked symptom relief following vestibular rehabilitation. This case underscores the importance of a thorough diagnostic workup and individualized treatment strategy in patients with complex vestibular presentations. Further evaluation with high-resolution computed tomography and ophthalmologic consultation is recommended to confirm the underlying pathology and guide ongoing care.

## Introduction

Persistent dizziness is a common yet diagnostically challenging complaint in clinical practice, often associated with a wide range of differential diagnoses that include vestibular, neurological, and systemic causes. Benign paroxysmal positional vertigo (BPPV), with an estimated lifetime prevalence of 2.4%, is one of the most common vestibular disorders, characterized by brief episodes of vertigo triggered by changes in head position. Superior semicircular canal dehiscence (SSCD), a less common but important differential, affects approximately 0.5% of the general population based on radiologic studies and involves a defect in the bony covering of the superior semicircular canal [1-4]. This opening can cause the inner ear to become unusually sensitive to certain stimuli, resulting in Tullio's phenomenon, a condition in which loud noises or pressure changes provoke symptoms such as vertigo, dizziness, or imbalance. Although rare, Tullio's phenomenon is most commonly seen in SSCD or other disorders that disrupt the normal structure of the inner ear. Intracranial hypertension, while even less common in the context of vestibular disorders, may contribute to symptoms through increased intracranial pressure and its effects on the optic nerves and surrounding structures [2,3]. This case report presents a patient with overlapping clinical features of these conditions, initially misdiagnosed and treated without improvement. The case illustrates the importance of a comprehensive, multidisciplinary approach, including advanced imaging and vestibular assessment, in identifying complex vestibular pathologies and optimizing patient outcomes.

## Case presentation

A 45-year-old Hispanic female with a history of hypertension presented with a two-week history of acute episodic dizziness described as a spinning sensation that worsened over the past few days. Her symptoms were exacerbated by loud noises and accompanied by left-sided ear pain, tinnitus, headaches, and intermittent blurred vision. She also reported experiencing right-sided esotropia, which she believed was a contributing factor to her visual disturbances. Before neurological evaluation, she was misdiagnosed with sinusitis and treated with a 10-day course of antibiotics and decongestants, which was discontinued due to excessive drowsiness and minimal symptom relief. Following referral, she was correctly diagnosed with Tullio's phenomenon.

The neurological evaluation included assessments of cranial nerve function, coordination, gait, and balance. A physical examination confirmed right eye esotropia and papilledema on funduscopy. The Dix-Hallpike maneuver elicited vertigo and bilateral rotary nystagmus with left-sided predominance when performed on the left side (Video 1).

**Video 1:** Bilateral rotary (torsional) nystagmus—left-predominant.

**Figure 1:**
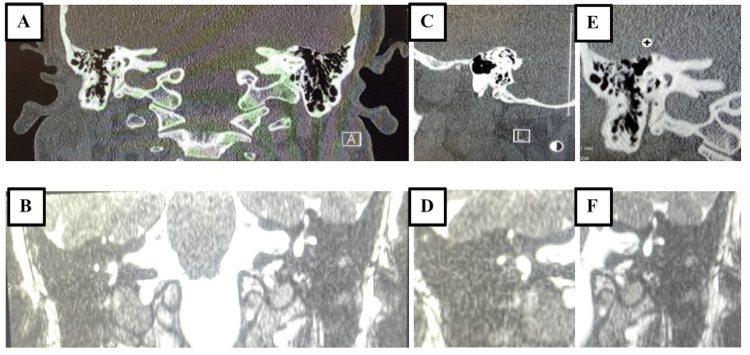
Bilateral superior semicircular canal dehiscence shown in coronal CT (A) and MRI (B). Right side zoom: CT (C), MRI (D). Left side zoom: CT (E), MRI (F).

Vestibular evoked myogenic potential (VEMP) testing showed lower thresholds and larger amplitudes, indicating increased sensitivity to sound and left-sided vestibular dysfunction. Audiometry revealed mild, low-frequency conductive hearing loss with an air-bone gap of approximately 25 dB at 250-1000 Hz, normal bone conduction thresholds, and normal middle ear pressure. However, the Rinne test was positive (air conduction greater than bone conduction), which typically suggests sensorineural hearing loss, while the Weber test lateralized inconsistently to the right ear. This paradox is characteristic of SSCD and results from a "third window" effect created by the dehiscent canal, which enhances bone conduction. Laboratory workup and EEG were normal. A brain MRI without contrast revealed bilateral thinning of the superior semicircular canals, raising suspicion for SSCD and the Tullio phenomenon (Figure 1). Additional findings included a partially empty sella turcica, optic nerve tortuosity, and papilledema, suggestive of intracranial hypertension.

Vestibular rehabilitation was initiated, focusing on habituation, gaze stabilization, and balance training. The program was carefully modified to avoid sound-induced symptom triggers, with exercises performed in a quiet environment without auditory stimulation for 20 to 30 minutes daily, divided into two to three sessions over approximately 8-12 weeks. Gaze stabilization (VOR ×1) exercises began with low-amplitude head movements, and balance tasks were gradually progressed based on tolerance. This individualized approach aimed to promote central compensation while minimizing the provocation of sound-sensitive vertigo. Over time, the frequency and severity of her symptoms gradually improved.

## Discussion

Tullio's phenomenon is a rare condition characterized by sound-induced vestibular symptoms, including dizziness, vertigo, imbalance, or nystagmus [1,2]. It arises from an abnormal interaction between the auditory and vestibular systems, which together regulate balance and spatial orientation [2,3]. One of the most common causes of Tullio's phenomenon is SSCD, a disorder caused by thinning or absence of the bony covering over the superior semicircular canal in the temporal bone. This defect creates an abnormal third mobile window in the inner ear, disrupting normal fluid dynamics and increasing sensitivity to sound and pressure changes. The abnormal opening enhances bone conduction at low frequencies, leading to conductive hearing loss on audiometry but a falsely positive Rinne test [1,2], as observed in this patient.

Patients with SSCD may present with a combination of auditory and vestibular symptoms, including sound- or pressure-induced vertigo (Tullio's phenomenon), autophony, oscillopsia, and low-frequency conductive hearing loss. In this case, SSCD was identified as the primary cause of the patient's sound-sensitive vertigo and imbalance [2]. Other potential causes, such as perilymphatic fistula and vestibular migraine, were considered less likely due to the absence of trauma or barotrauma and no history of migraines. Diagnosis relies on a detailed clinical history, audiological and vestibular testing, and high-resolution CT imaging of the temporal bones to confirm anatomical abnormalities, such as SSCD [1-3]. Management ranges from conservative strategies such as trigger avoidance, vestibular rehabilitation, and symptom control to surgical repair in cases with confirmed structural defects like SSCD or perilymph fistula [2,4].

## Conclusions

This case underscores the complexity of diagnosing vestibular disorders, particularly in patients with overlapping neurological and auditory symptoms. The clinical presentation, combined with physical examination, imaging, and laboratory findings, suggests a multifactorial vestibular pathology with possible contributions from intracranial hypertension and SSCD. Although a definitive diagnosis remains to be established, other potential differentials, such as perilymphatic fistula and vestibular migraine, were considered less likely based on the absence of trauma, barotrauma, or migraine history. This highlights the diagnostic challenges in distinguishing between structural, vascular, and idiopathic causes of dizziness.

The patient's significant improvement with vestibular rehabilitation emphasizes the value of individualized, noninvasive therapeutic strategies. A tailored program targeting symptom triggers led to reduced frequency and severity of symptoms over time. Further diagnostic refinement through high-resolution temporal bone CT and formal ophthalmologic evaluation is warranted to confirm underlying anatomical abnormalities and guide long-term management. Ultimately, this case reinforces the importance of a comprehensive, multidisciplinary approach, including detailed history-taking, focused physical examination, and advanced imaging in the evaluation and treatment of persistent and unexplained dizziness.

## References

[1] Lehmkuhl B, Andaloro C (2022). Tullio phenomenon. StatPearls [Internet].

[2] Kaski D, Davies R, Luxon L, Bronstein AM, Rudge P (2012). The Tullio phenomenon: a neurologically neglected presentation. J Neurol.

[3] Iversen MM, Zhu H, Zhou W, Della Santina CC, Carey JP, Rabbitt RD (2018). Sound abnormally stimulates the vestibular system in canal dehiscence syndrome by generating pathological fluid-mechanical waves. Sci Rep.

[4] Suzuki M, Ota Y, Takanami T, Yoshino R, Masuda H (2024). Superior canal dehiscence syndrome: a review. Auris Nasus Larynx.

